# Clinical outcomes of xenogeneic acellular dermal matrix combined with full-thickness skin graft in the eyelid reconstruction following the excision of eyelid malignant tumors

**DOI:** 10.3389/fonc.2025.1627742

**Published:** 2025-07-09

**Authors:** Linlin Xie, Jing Xie, Wei Wang

**Affiliations:** ^1^ The First Clinical Medical College of Gannan Medical University, Ganzhou, Jiangxi, China; ^2^ Department of Ophthalmology, The First Affiliated Hospital of Gannan Medical University, Ganzhou, Jiangxi, China; ^3^ Xiamen Medical College Affiliated Haicang Hospital, Xiamen, Fujian, China

**Keywords:** xenogeneic acellular dermal matrix, full-thickness skin graft, eyelid reconstruction, surgical approach, eyelid malignant tumors

## Abstract

**Objective:**

This study investigates the clinical efficacy of using the xenogeneic acellular dermal matrix (XADM) combined with full-thickness skin graft (FTSG) to reconstruct full-thickness eyelid defects following the excision of large malignant tumors.

**Methods:**

This single-center retrospective observational study analyzed 21 patients who received XADM combined with FTSG for full-thickness eyelid defects reconstruction following tumor resection at our hospital between January 2020 and September 2022. The study was reported according to the STROBE statement reporting guidelines. The cases included 13 basal cell carcinomas, one meibomian gland carcinoma, and seven squamous cell carcinomas. In the first-stage surgery, XADM combined with a FTSG was used to reconstruct the full-thickness eyelid defect. In the postoperative, a pressure dressing was applied for 5 days removing stitches at 2 weeks. Repair outcomes were assessed at 1, 3, and 6 months postoperatively. At the 6 months postoperative follow-up, second-stage surgery was performed to separate the upper and lower eyelids, restoring normal eyelid anatomy and function.

**Results:**

At 2 weeks postoperatively, all graft marginal sutures were well-aligned, and no graft necrosis was observed after sutures were removed. No complications were found during the follow-up at 1 and 3 months postoperatively. At 6 months postoperatively, separation of upper and lower eyelid adhesions was performed. Slit-lamp examination post eyelid separation surgery reveals the overall healing outcome was satisfactory, with no significant inflammatory response or rejection observed. The overall healing outcome was satisfactory, with no significant inflammatory response or rejection observed. All 21 patients recovered well postoperatively. No significant complications were observed in any patient, with only one patient exhibiting mild lagophthalmos.

**Conclusion:**

In full-thickness eyelid reconstruction following the excision of eyelid malignant tumors, using XADM for reconstructing the posterior lamella and conjunctiva, combined with FTSG for repairing the eyelid skin and soft tissue, is a recommended approach.

## 1 Introduction

The eyelid covers the anterior surface of the eyeball and serves several primary functions, including protection of the globe, participation in tear secretion and reduction of tear evaporation, maintenance of the ocular surface environment, and aesthetic enhancement. Full-thickness eyelid defects may lead to secondary complications such as lagophthalmos and ocular exposure (1). They not only compromise the morphology and function of the eyelid skin but also affect the patient’s vision and appearance (2). Therefore, eyelid reconstruction not only reduces the risk of complications associated with eyelid defects but also improves ocular function and aesthetic outcomes for the patient.

Eyelid reconstruction is a functional treatment that repairs or rebuilds the structure of the eyelid through surgery. The goal is to restore both functionality and aesthetic outcomes using a minimally invasive approach while preserving the ocular surface environment and preventing damage to visual function (3, 4). To preserve these functions, eyelid reconstruction should be planned according to the principles of similarity and the reconstructive ladder. Depending on the extent and severity of the defect, tissues that are proximate or similar to the defect site should be employed to restore both functional and cosmetic aspects (5).

XADM is a biological material that can be selected for the reconstruction of the posterior lamella of the eyelid which is made from animal skin that has undergone decellularization. This process removes cellular components while retaining matrix components such as collagen, making it highly biocompatible. XADM provides a scaffold for repair, promotes soft tissue regeneration, and reduces scar formation (6).

This study aimed to reflect the observational outcomes following full-thickness eyelid reconstruction using XADM combined with FTSG after resection of medium-to-large malignant eyelid tumors, including graft integration, restoration of eyelid anatomy, incidence of complications, etc. We also summarizes real-world data to enrich the experience and optimize surgical techniques. In recent years, we have successfully treated 21 cases of full-thickness eyelid defects following the resection of malignant eyelid tumors by combining XADM with FTSG. The outcomes were favorable, and the analysis is reported as follows.

## 2 Clinical data and methods

### 2.1 Patients data

Our study included 21 patients (21 eyes) with eyelid malignancies treated at the First Affiliated Hospital of Gannan Medical University between January 2020 and September 2022. The cohort consisted of 6 males (6 eyes) and 15 females (15 eyes), with ages ranging from 53 to 83 years (mean age: 68 years). Among them, eight cases involved the upper eyelid and 13 cases involved the lower eyelid. Pathological diagnoses included 13 cases of basal cell carcinoma, one case of meibomian gland carcinoma, and seven cases of squamous cell carcinoma. Post-tumor resection, significant eyelid involvement was observed, with defects exceeding 1/2 of the eyelid in 6 cases and ranging between 1/3 and 1/2 in 15 cases (**Table 1** for details).

**Table 1:** Basic information and clinical characteristics of patients.

Pathological type	Males	Females	Left eye	Right eye	Upper eyelid	Lower eyelid	Defect eyelid area between 1/3-1/2	Defect eyelid area >1/2
Basal cell carcinoma	8	5	8	5	3	10	9	4
Squamous cell carcinoma	6	1	3	4	4	3	5	2
Meibomian gland carcinoma	1	0	1	0	0	1	1	0

### 2.2 Methods

The surgical material used was the xenogeneic dermal matrix Heal-all Oral Biofilm (size: 2 cm × 2.5 cm), provided by Yantai Zhenghai Bio-Technology Co. Ltd. The surgical procedures included eyelid tumor resection, bioengineered membrane transplantation, FTSG transplantation, and eyelid reconstruction. All surgeries were performed under general anesthesia with the patient in the supine position.

### 2.3 Surgical procedure

**Figure 1:**
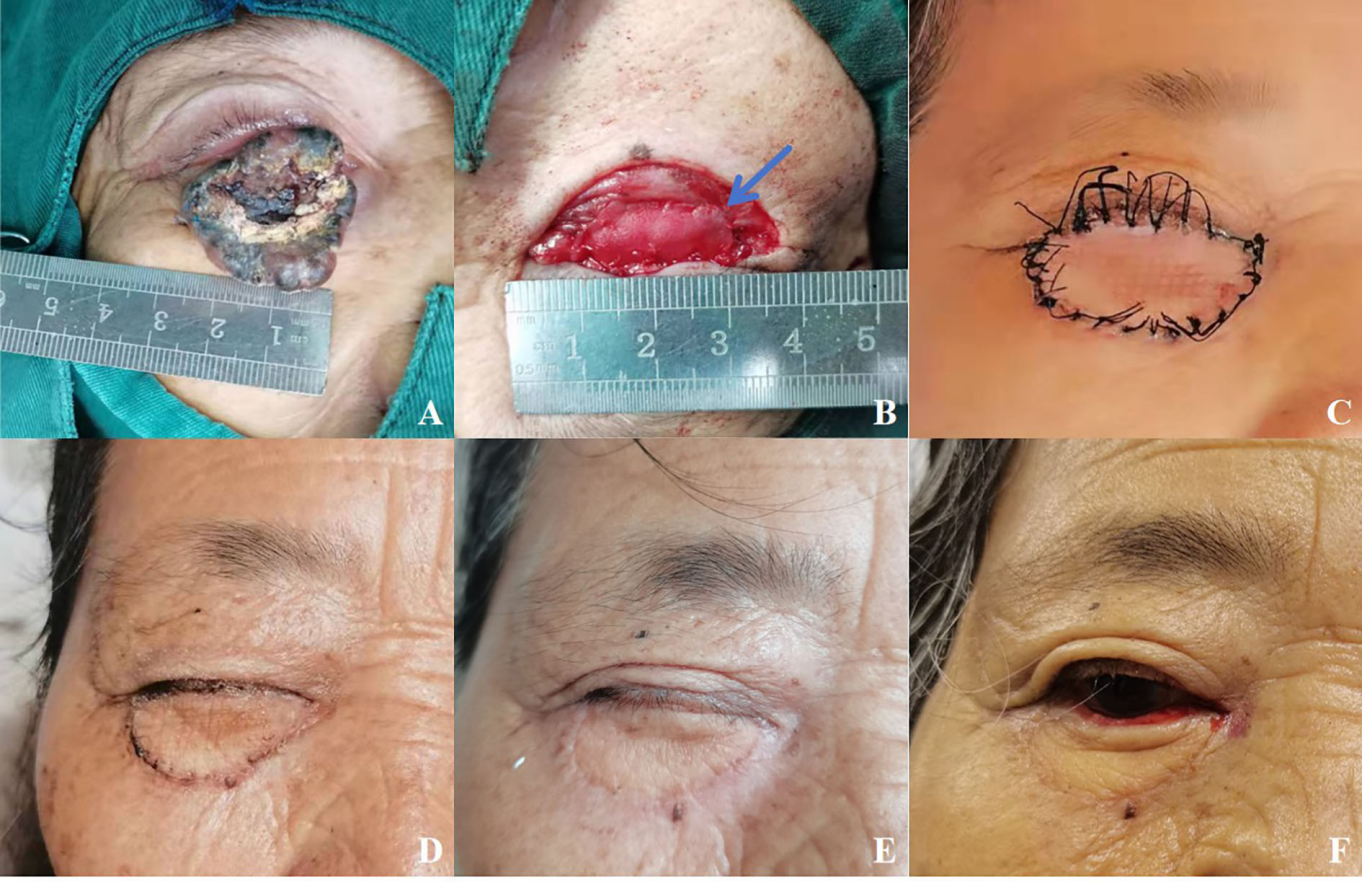
**(A)** A patient with basal cell carcinoma of the right eye, female, 68 years old. **(B)** The eyelid defect was more than 2/3. The lower tarsal plate (blue arrow) was replaced by the XADM. **(C)** 2 weeks postoperatively before the sutures are removed. **(D)** Postoperative appearance at 1 month after eyelid reconstruction. **(E)** Upper and lower eyelids were adherent postoperatively. **(F)** Postoperative appearance at six months after eyelid reconstruction.

**Figure 2:**
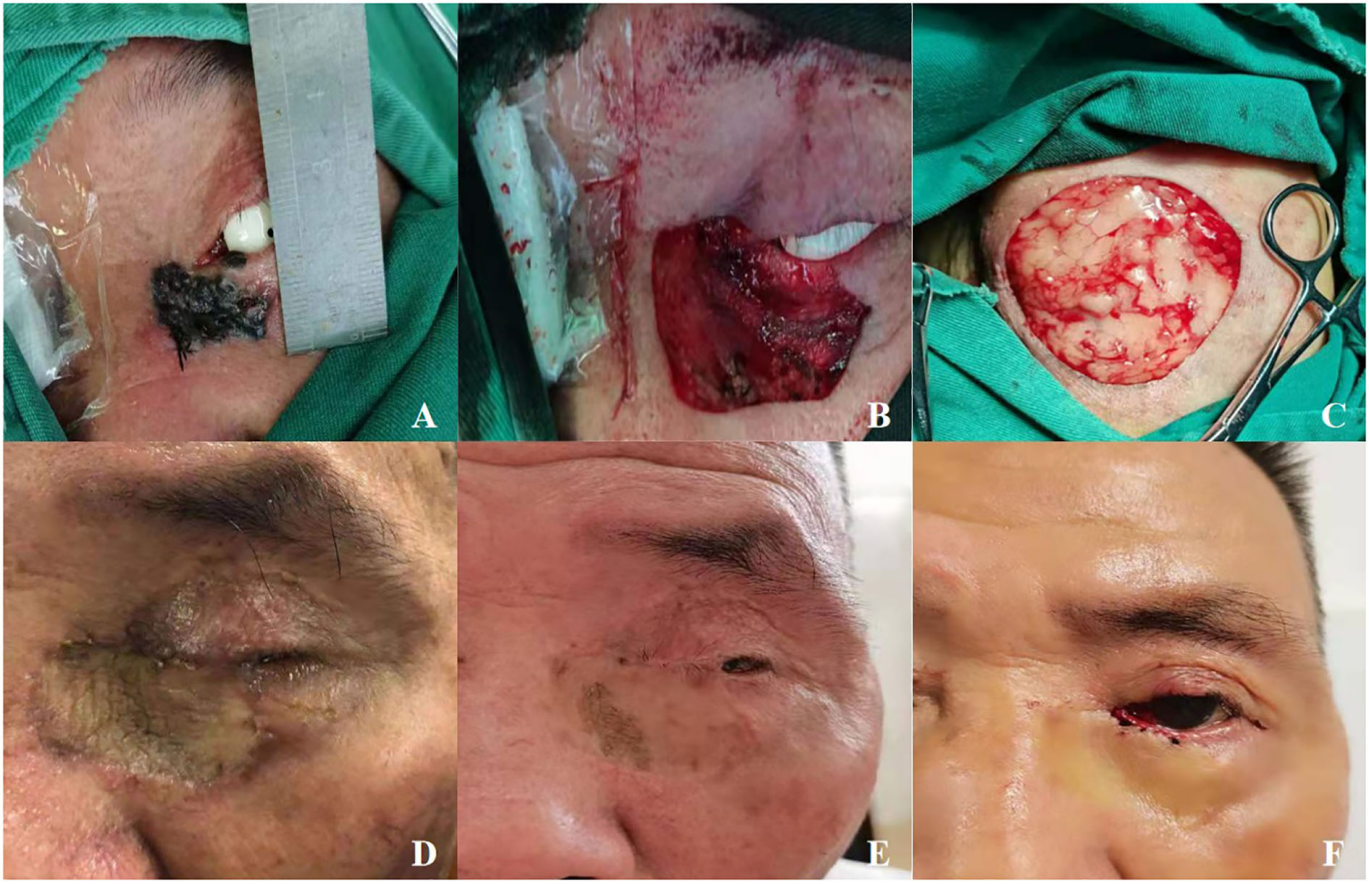
**(A)** A patient with squamous cell carcinoma of the left eye, male, 75 years old. **(B)** Image B displays the area after lesion removal. **(C)** Harvested a skin graft from the lax skin of the patient's forearm. **(D)** 1 month after eyelid reconstruction. **(E)** Upper and lower eyelids were adhered postoperatively. **(F)** 9 months postoperative aspect after eyelid reconstruction.

1. Traditional wide excision: after anesthesia administration, the approximate resection area was marked using methylene blue. The incision was made 2**–**5 mm from the tumor margin, ensuring as smooth resection margins as possible. The tumor was marked and divided into small with margins preserved to ensure the localization of residual lesions, then rapidly frozen and sent to the pathology department. Technical method for frozen section preparation: (1). The margin tissue was sliced into thin sections using a microscope, stained, and examined under a microscope. (2). The entire process typically takes 30**–**45 minutes to have a diagnosis. (3). Vertical sections were employed to sample partial margins. In all cases, the resected tissues were sent for intraoperative frozen section pathological examination. If the pathological examination indicates incision margin involvement, the wider excision was performed concurrently. Following tumor resection, full-thickness eyelid defects of varying sizes were observed.
2. An XADM bioengineered membrane was prepared to reconstruct the posterior lamella. Firstly, the severed ends of the tarsal plate at the defect site should be trimmed into a “stepped” or “wedge-shaped” configuration to ensure a smooth eyelid margin. The XADM is sized to match the defect and securely anchored approximated with interrupted 6–0absorbable sutures to the residual tarsus. If the defect area is close to the canthus, the XADM should be anchored to the medial and lateral canthal tendon to prevent displacement (**Figure 1B**).
3. Following lesion resection, FTSG transplantation was performed for cases with extensive defects. Before the transplantation, the size of the defect was estimated and measured.
4. A skin graft of comparable size to the eyelid defect was harvested from the lax skin of the patient’s forearm (**Figure 2C**). The skin was incised down to the junction between the full dermal layer and subcutaneous fat tissue, ensuring complete harvest of both epidermis and full-thickness dermis while minimizing fat inclusion and maintaining optimal graft thickness. The harvested FTSG was placed in a saline solution. Any incidentally harvested subcutaneous fat is subsequently removed and the edges of the graft are then trimmed to ensure a smooth margin.
5. The skin graft was positioned along the margins of the eyelid defect. Simultaneously, adequate relaxation of the surrounding tissue in the eyelid defect area is performed to reduce tension and promote postoperative wound healing.
6. The FTSG was secured to the eyelid defect area using the bolster sutures technique with 3**–**0 silk sutures.
7. The eyelid margin gray line was incised, and the anterior and posterior lamellae of the gray line were excised to create a wound. The residual tarsal plates of the upper and lower eyelids were then sutured to the skin graft using non-absorbable sutures (6**–**0 silk) in apposition sutures. Place the vaseline gauze (**Figure 1C**, with corticosteroid ointment applied on its inner surface) and cotton balls in the specified order from inside to outside. Secure the dressing using the sutures from the full-thickness skin graft fixation. Suture fixation method: First, divide the sutures into four sections along four directions, gathering all sutures in each section into a bundle. Then, tie the opposing bundles together pairwise for fixation.
8. Apply a bandage to the head to further fix the dressing over the grafted area.
9. For the forearm donor site management: primary wound closure was performed using 3**–**0 silk sutures, followed by gauze coverage to prevent infection.

### 2.4 Postoperative management

1. Starting from the first day after surgery, the operated eye is maintained under continuous pressure bandaging. Prophylactic systemic antibiotics were administered for 24 hours. To patients who report more severe postoperative pain, oral nonsteroidal anti-inflammatory drugs are administered to relieve postoperative pain. The operated eye was continuously bandaged for 5 days, Remove the gauze daily and gently clean the dressing ointment at the margin of the grafted area with saline under aseptic technique. Observe the grafted area through the wound edges, then replace a small portion of the cotton balls dressing and reapply the bandage in the outer.
2. Key observation on the third day after surgery to assess skin graft survival status. Including the blood supply of the graft area, the presence of exudation, and any signs of infection, while purple-black or “leather-like” appearance signaled potential ischemia or necrosis.
3. From the fifth day after surgery, remove the bandage while retaining the dressing and sutures. Order the patient to monitor the grafted area, perform iodine solution disinfection in the sutured margins of the graft daily, and keep the graft site dry.
4. 2 weeks postoperatively, the pressure dressing and skin sutures on the FTSG were removed. Outpatient follow-up evaluations were conducted at 1, 3, and 6 months postoperatively. At the 6-month postoperative follow-up, second-stage surgery was performed, and the fixation sutures between the residual upper and lower tarsal plates were removed simultaneously to restore normal eyelid anatomy and function. Under local anesthesia and aseptic conditions, the sutures were removed using ophthalmic forceps and scissors. The around suture tissue was examined for signs of granuloma formation or infection. If suture breakage or residue was present, complete removal was ensured under a microscope. Focus on evaluating the integration status of the XADM and FTSG: During the follow-up, the healing and repair of the FTSG and the XADM were monitored, with particular attention to signs of graft separation, necrosis, or exposure, and the graft site was evaluated for skin color, signs of infection, skin temperature, tension, and the presence of foreign body sensations (**Figures 1D**, **2D**). Additionally, the patient’s systemic condition was monitored for any adverse reactions.

## 3 Results

At 6 months postoperatively, the upper and lower eyelid margins were separated, followed by systematic evaluation and documentation of patient recovery outcomes. The graft survival rate in this cohort was 100%. Slit-lamp examination post eyelid separation surgery reveals: that the grafted skin demonstrates excellent approximation, with well-aligned wound edges showing no significant displacement or elevation. The recipient bed remains dry and clean, without evidence of bleeding, exudate, or discharge accumulation. The skin graft exhibits showing no pallor or other signs of necrosis. Eyelid closure function is complete, with normal marginal position. No cases of tumor recurrence or metastasis were observed during follow-up. The incision scars were inconspicuous. 20 patients had good recovery of eyelid morphology, with no significant displacement or ectropion/entropion. One patient exhibited mild lagophthalmos, specifically manifested as the inability of the eyelids to cover the eyeball when closed eyes completely. This condition may result from inadequate tissue reconstruction, scarring, contracture, or patient-specific factors (7). No patients developed exposure keratitis.

## 4 Discussion

Studies indicate characteristics of eyelid reconstruction surgery that include the following: (1) applicability to eyelid defects caused by various etiologies; (2) absence of conjunctival and corneal irritation; (3) excellent functional and appearance restoration (8, 9). The chosen approach for eyelid reconstruction is based on the specific defect and patient characteristics, such as using ear cartilage strip graft and oral mucosa graft to reconstruct posterior layer eyelid defects (8) and so on. The strategy of reconstruction is formulated based on a comprehensive assessment of the defect location, extent, and condition of surrounding periocular tissues. The use of skin grafts and tissue flaps for reconstructing cutaneous or myocutaneous defects of the eyelid has become a well-established technique, achieving satisfactory functional and aesthetic results in clinical practice. During eyelid reconstruction, direct suturing can be selected for small defects. For small to medium-sized defects, adjacent skin graft transplantation and local tissue flap transplantation are suitable for repair. However, for larger eyelid defects, the limited availability of facial skin makes adjacent skin graft transplantation unsuitable (10).

Eyelid defects can be categorized by layer of involvement into superficial defects, deep-layer defects, and full-thickness defects; classified by anatomical location into upper eyelid defects, lower eyelid defects, medial canthus defects, or lateral canthus defects; classified according to the degree of severity: mild defects (involving less than 1/3 of the eyelid margin length), moderate defects (involving 1/3 to 1/2 of the eyelid margin length), or severe defects (involving more than half of the eyelid margin length) (1). When the eyelid defect is less than 1/4 of the eyelid size (in young patients) or 1/3 (in elderly patients), it can be repaired by direct suturing. For defects involving 1/3 to 1/2 of the eyelid size, local flaps (such as a Tenzel flap) or grafts (such as a tarso-conjunctival graft) are required for reconstruction.

In cases of larger defects exceeding 1/2 of the eyelid size, more complex flap techniques (such as the Hughes flap) or composite grafts (such as cartilage-mucosal grafts) are required for layered reconstruction (1). In this study, all cases sustained full-thickness eyelid defects encompassing over 1/3 of the palpebral surface area following surgical excision of malignant eyelid tumors (**Table 1** for details). Therefore, a layered reconstruction of the eyelid defect was required, with the restoration of both the anterior and posterior lamellar functions. Our study’s methodology is particularly suitable for patients with eyelid defects involving more than 1/3 of the eyelid, especially those with full-thickness defects exceeding 1/2 of the eyelid. For defects larger than 1/2, the Tenzel flap shows limited applicability due to tension constraints, while Hughes flap repair requires additional tissue to reconstruct the anterior lamella. In contrast, our approach provides sufficient tissue volume to cover larger defect areas while maintaining the natural appearance and function of the eyelid. Compared with conventional methods, our results demonstrate a lower complication rate.

Currently, the commonly used reconstruction materials for different anatomical locations of defects include: (1)Anterior lamellar defects: FTSG and local flaps; (2)Posterior lamellar defects: (a) Autologous tissue: Including periosteal flaps (11), conjunctival grafts, oral mucosal grafts, cartilage grafts, and tarso-conjunctival grafts. (b) Allogeneic tissue: Such as allogeneic oral mucosa, allogeneic conjunctival grafts, etc. (c) Bioengineered materials: XADM, etc. (d) Synthetic materials: polytetrafluoroethylene, silicone, etc. (3)Full-thickness defects: XADM, complex flaps (Hughes flap, Tenzel flap), composite graft biomaterials (Boston Keratoprosthesis Type II) and so on (12–16). Grafts used for repairing the eyelid skin and palpebral conjunctival surface should exhibit the following characteristics: easy accessibility, appropriate thickness, high maneuverability, and low risk of rejection. Currently available tarsal plate substitutes include: autologous hard palate mucosa (7), autologous auricular and nasal septal cartilage (8), allogeneic sclera1, XADM, and synthetic biomaterials (e.g., Boston Keratoprosthesis Type II).

XADM is used in several clinical disciplines, including burn (17), stomatology (18) and surgical oncology (17). Many studies have demonstrated that, compared with traditional autologous tissues, XADM exhibits significant advantages and efficacy in reconstructing eyelid defects. This material retains the non-antigenic extracellular matrix components, which provide a three-dimensional scaffold structure with collagen fibers for tissue growth. This allows the fibroblasts and collagen fibers to arrange in a regular and structured manner during the proliferation process, facilitating tissue remodeling and thus improving wound healing (19). During the production of XADM, the xeno cells that cause the immune response are removed and effectively reduce antigenicity, rejection, and inflammation (20, 21). XADM exhibits excellent mechanical properties and sufficient rigidity to support the eyelid. Additionally, it can induce connective tissue growth in the recipient site, thereby preventing graft absorption. These characteristics contribute to the restoration of both functional and aesthetic outcomes, particularly in cases of full-thickness eyelid defects (22). Various studies have demonstrated that XADM offers numerous advantages in repairing tissue defects, including low risk of rejection, wide availability of sources, cost-effectiveness, versatile sizing options, optimized surgical procedures, and low incidence of postoperative complications. Given these benefits, it has become a popular choice for reconstructing eyelids, conjunctiva, and conjunctival sac defects (23, 24).

In this study, the majority of patients with malignant eyelid tumors were elderly, with a prolonged disease course and extensive tumor involvement of the eyelid skin. Consequently, the eyelid defects following tumor resection were often large. The use of adjacent flap transposition may cause postoperative skin contracture, subsequently resulting in lagophthalmos and bilateral asymmetry, which can impair both functional outcomes and aesthetic results. Additionally, patients might require additional surgeries to address complications such as local scar laxity. Reasons analyzed according to the above discussion, we employed FTSG transplantation to repair significant eyelid defects in this study. This technique was introduced to Europe in the 19th century (25) and has been widely adopted in repairing defects caused by trauma or tumor resection in disciplines such as oral and maxillofacial surgery and surgical oncology due to the advancement of microsurgical techniques. The use of FTSG for eyelid skin defects has the following advantages: (1)The color and thickness of the FTSG are closely consistent with the eyelid skin, providing excellent pliability and aesthetic outcomes; (2)The forearm offers a large donor area with a relatively simple structure, making it easier to harvest graft for larger eyelid defects; (3) It has a high survival rate and fewer complications (26). (4) The forearm region has abundant blood supply, which facilitates postoperative donor site recovery, particularly in elderly patients with diminished tissue repair capacity.

This study employed a reconstructive method combining FTSG with XADM. By integrating the versatility of skin graft and the biocompatibility and tissue integration of XADM, this approach can effectively restore the function and appearance of the eyelid. The advantage of this method lies in providing a sufficient amount of tissue to cover larger defects while maintaining the natural appearance and function of the eyelid.

Moreover, compared to traditional eyelid surgery techniques, real-world data demonstrate that our method results in fewer postoperative complications. Common complications following eyelid reconstruction include ectropion, wound dehiscence, functionally acceptable but visible scarring, and retraction, among others (27). By combining surgical techniques with postoperative management strategies, we effectively prevent complications such as ectropion. Specific measures include: (1) Structural Support Techniques: Graft size matching the defect area to avoid excessive tension, Reinforcement suturing techniques. (2) Biomaterial Selection: Use of full-thickness skin grafts combined with XADM to ensure optimal postoperative eyelid stability, leveraging appropriate tensile strength. (3) Postoperative Management: Continuous pressure dressing for the first 5 days post-surgery. Secondary eyelid incision at 6 months to prevent premature separation and subsequent ectropion.

Although this study has achieved certain results in evaluating the eyelid reconstruction technique combining XADM with skin graft from the forearm, there are still some limitations. First, the six-month fusion period of the upper and lower eyelids represents our carefully considered balance between providing adequate healing time and minimizing patient implications. But it also represents a significant limitation of this technique: (1) Implications on patient quality of life: Prolonged time for the fusion significantly restricts patients’ vision and stereopsis, potentially affecting daily activities. The appearance may also cause psychosocial distress. (2) Risk of complications: While the fusion protects the cornea and allows extended healing time for the reconstruction site, it may also increase risks of fibrosis, adhesions, and functional impairment. Second, the sample size limits the statistical analysis of the results and the generalizability of the conclusions. Third, the 6-month postoperative follow-up period is not sufficient for assessing long-term function and aesthetic outcomes, there is a lack of standardized functional assessment scales, a lack of vascular imaging data for the reconstructed transplant area, etc. Finally, despite showing good biocompatibility and tissue integration in short-term follow-ups, the long-term effects of XADM still need further research.

## 5 Conclusion

Extensive full-thickness eyelid defect reconstruction is challenging and requires a thorough understanding of eyelid anatomy and repair techniques. These defects commonly occur after resection of malignant eyelid tumors, and successful outcomes depend on the defects and reconstruction techniques. This study summarized and analyzed the reconstruction method using XADM combined with FTSG for full-thickness eyelid defects after malignant tumor excision. The surgical method of using XADM to reconstruct the posterior layer (tarsus and conjunctiva) and FTSG to repair the eyelid skin and soft tissue can effectively achieve eyelid reconstruction. This method has significant advantages, including fewer short-term complications and stable recovery of eyelid function and appearance. Based on our results, we recommend the use of this method for patients with full-thickness eyelid defects that exceed 1/3 of the eyelid, especially those exceeding 1/2, as it can achieve excellent eyelid reconstruction clinical outcomes.

## Data Availability

The original contributions presented in the study are included in the article/supplementary material. Further inquiries can be directed to the corresponding author.
